# Virchows Archiv (The European Journal of Pathology): Past and future

**DOI:** 10.1007/s00428-023-03498-6

**Published:** 2023-01-24

**Authors:** Abbas Agaimy

**Affiliations:** Institute of Pathology, University Hospital Erlangen, Friedrich‐Alexander University Erlangen‐Nürnberg (FAU), Erlangen, Germany

Dear readers,

In the year 2006, I have published with great enthusiasm my first paper in Virchows Archiv [1]. Since then, my name was listed as author/coauthor in 40 papers published in the journal, one of the most recent of them defining a novel entity has made its direct short-cut way from Virchows Archiv into the WHO classification [2, 3]. My experience as an author was mirrored for several years by a regular activity as a reviewer and editorial board member. During this period, I have developed a feeling of high loyalty and commitment to the journal and a deep insight as to the type of manuscripts much welcomed by the journal editors. However, it was not imaginable for me to expect such a quick game change from an author and a reviewer to an associate editor during 2021-2022 and an Editor-in-Chief starting 2023.

Having that said, I am most thankful to the European Society of Pathology (ESP) and Springer for trusting me to overtake the challenging and demanding role of the new Editor-in-Chief of Virchows Archiv. With a mixed feeling of honor and privilege, enthusiastic commitment, and loyalty, I appreciate and admit, how difficult this task will be, when considering the great effort and the tremendous work the past editors of the journal since the union of its A and B sections have put forward, to insure such a successful development of our journal. The evolving future plans and the implemented great work, which became more visible during the editorship of Fred Bosman [4] and Daniela Massi [5], made Virchows Archiv to one of the most read and influential journals in our discipline, no need to mention the significant increase in the downloads of articles, the rising impact factor and other journal metrics.

During my Chief Editorship term, my goal will be to maintain the established line and high standard of Virchows Archiv, at same time trying to make the unification of the historical A and B sections of the journal more visible in each published article. Accordingly, the types of articles we wish to receive and publish should contribute to our understanding of the scientific basis and the complex mechanisms underlying human diseases including molecular and translational studies, so fulfilling the goals of the historical section B of the journal (*Cell Pathology*). At same time, these studies should have a significant positive impact on the practice of our discipline in one of its three major pillars: diagnostics, prediction and prognosis, thereby contributing to the improvement in patient’s care, the ultimate goal of our job. Hence, a major feature of acceptable articles is that they should unequivocally positively influence the practice of modern diagnostic and predictive surgical pathology in line with the historical section A of the journal (*Pathological Anatomy and Histology*).

Besides the annual review issue, my aim will be to have at least one major review article published in each issue addressing timely hot topics related to *emerging tools*, *emerging concepts, controversies*, *pitfalls,* and *perspectives in surgical pathology*. Moreover, special reviews will be devoted to *best practice recommendations immunohistochemistry* in surgical pathology as well as timely *updates* on the most recently published WHO “blue” books. ESP position papers guided by the ESP working groups, experts’ opinion papers, and joint guidelines developed by the ESP in collaboration with different clinical societies will be much encouraged. Last but not least, timely topics on non-neoplastic diseases at the intersection between diagnostic pathology and clinical medicine, including in particular original studies and review articles dealing with pathological conditions related to recently approved or emerging new targeted therapies as well as state-of-the-art diagnostics of inflammatory (infectious and autoimmunological) diseases are much welcome.

Finally, no need saying, that the great success the journal has made and the anticipated further development of our journal would haven´t been and wouldn´t be possible without the tremendous joint efforts of our editorial board, the associate editors and the (much appreciated) demanding work by our reviewers; all being inevitable for maintaining the high-quality scientific contents which became the standard in each published issue of Virchows Archiv.

Abbas Agaimy, M.D.

Editor-in-Chief
